# Probing the dispersive properties of optical fibers with an array of femtosecond-written fiber Bragg gratings

**DOI:** 10.1038/s41598-022-08329-3

**Published:** 2022-03-14

**Authors:** Tommy Boilard, Réal Vallée, Martin Bernier

**Affiliations:** Center for Optics, Photonics and Lasers (COPL), Université Laval, Québec, QC G1V 0A6 Canada

**Keywords:** Fibre optics and optical communications, Mid-infrared photonics, Fibre lasers, Ultrafast lasers, Optics and photonics, Solitons, Supercontinuum generation, Ultrafast photonics, Characterization and analytical techniques

## Abstract

We propose an efficient method to determine the effective refractive index of step-index optical fibers from the visible to the mid-IR and thus allowing to infer their dispersive properties over a broad spectral range. The validity of the method, based on the writing of an array of fiber Bragg gratings (FBGs) with known periods using the fs scanning phase mask technique, is first confirmed with a standard silica fiber, then applied to various fluoride glass fibers to determine their effective refractive index and dispersion over more than three octaves, i.e. from 550 to 4800 nm.

## Introduction

There has been an increased interest in the last decade toward the development of pulsed fiber sources operating in the mid-infrared (mid-IR). Numerous applications are indeed calling for bright and powerful laser sources with excellent beam quality and covering the so-called molecular fingerprint region. In molecular spectroscopy and environmental sensing, for example, several atmospheric pollutants including carbon oxides (CO, CO_2_), hydrocarbons such as methane (CH_4_) and nitrogen oxides (NO_X_), possess absorption lines in the 3–5 μm spectral region that can be 10 to 100 times stronger than their respective harmonics lying in the near-infrared (near-IR)^1^. This spectral region also happens to be an atmospheric transparency window, allowing for the remote sensing of various molecules over long distance^2^.

Mid-IR ultrafast fiber sources based on direct light generation within femtosecond fiber laser^3–6^, or based on soliton self-frequency shift (SSFS)^7,8^ and supercontinuum generation (SC)^9–13^ closely rely on the nonlinear and dispersive properties of the optical fibers to operate. Knowing, and eventually being able to precisely tailor, the shape of a dispersion curve over a broad spectral range extending up to the limit of transparency of the fiber material, is especially crucial for the optimization of such mid-IR sources. Now, although the physical properties, especially the refractive index, of silica fibers are now rather well known, those of fiber materials with extended transmission in the mid-IR, e.g. fluoride [including fluorozirconate (ZrF_4_) and fluoroindate (InF_3_)], tellurite and chalcogenide glasses are far less documented.

Actually, the refractive indices of mid-IR fibers are either theoretically inferred from their glass compositions^14^, or directly measured from the fibers^15^ or from bulk materials with a prism spectrometer, the validity of the latter being uncertain due to the decrease of the refractive index of the fiber upon drawing^16^. Other means exist for directly determining the dispersion of optical fibers, such as by inferring it from the Kelly side lobes associated with ultrafast pulses propagating inside a ring cavity^3^ or by using complex or dedicated interferometric setups^17,18^.

Another method that has been applied to highly sensitive silica-on-silicon waveguides is to write an array of weak FBGs to infer their dispersion from the measurement of their effective refractive index from 800 nm to 1600 nm^19^. In silica optical fibers, this method has only been limited to a mere 50 nm spectral bandwidth^20^, and its demonstration on a larger scale or in non-photosensitive fiber has yet to be made.

In this paper, by writing an array of FBGs with precisely known periods using the femtosecond scanning phase mask technique, we accurately determine the dispersive properties of non-photosensitive specialty optical fibers over a bandwidth extending over more than three octaves, i.e. from the visible (550 nm) to the mid-IR (4800 nm). The approach consists in measuring the first, second and third diffraction orders of FBGs to retrieve the effective refractive index (${n}_{\mathrm{eff}}$) spectral distribution of the fiber’s fundamental mode, that is finally used to infer the dispersion of the fiber. Our results are first validated in a standard silica fiber (SMF-28, Corning), and then extended from the visible to the mid-IR in an erbium-doped fluorozirconate optical fiber and three undoped fluoroindate optical fibers. Finally, we propose to extend this method to characterize the dispersion of other exotic fibers and to characterize other optical and physical parameters of such fibers, namely their cutoff wavelength, their numerical aperture and even their core diameter by measuring the Bragg wavelength associated with higher order modes propagating in the fiber.

## Methods

### FBGs inscription

The fs scanning phase mask writing setup used for this experiment has been used extensively in the past decade to write strong and low losses FBGs through the protective coating of silica, chalcogenide, and fluoride glass fibers^21–23^ and was further optimized to efficiently write distributed arrays of FBGs for sensing applications^24,25^. In brief, a Ti:Sapphire amplifier (Astrella, Coherent) is used to generate 30 fs pulses at 800 nm with a repetition rate of 1 kHz. The pulses are tightly focalized with an acylindrical lens (f = 8 mm), through a phase mask, inside the core of an optical fiber. The period of the FBG is half the period of the phase mask, i.e. Λ = Λ_PM_/2. Due to the transparency of acrylate coatings at 800 nm, FBGs can easily be written through it, which is an important feature for writing in exotic, and sometimes brittle, optical fibers. Still, the most important feature of the writing setup for the present experiment is definitively the ability to write FBGs in any type of transparent material owing to the multi-photon absorption mechanism. To increase the overlap between the FBG and the mode inside the fiber, the lens is mounted on a three-axis piezoelectric actuator and is scanned over the core of the fiber, thereby increasing the FBG’s reflectivity. Finally, the lens and the beam are moved along the length of the fiber with a high precision translation stage to control the FBG length.

For this experiment, we designed a custom phase mask that was fabricated by e-beam lithography with twenty-four consecutive 5 mm-long uniform gratings with periods evenly distributed from 1000.0 nm to 3300.0 nm along its 120 mm length. Instead of standard uniform phase masks, the benefit of this phase mask is that multiple FBGs can be written inside a single strand of optical fiber without the need for changing and aligning the phase masks each time. Each period of the phase mask is selected by the position of the lens (i.e. the translation stage), and the FBGs are written one at a time with a multi-pass procedure to reach the targeted reflectivity. This procedure is adopted, instead of a single scan over the entire length of the phase mask at once, because a deterioration of the spectral quality of the FBGs was observed when the laser beam overlapped two adjacent gratings of the phase mask due to interference^26^. Also note that the shortest pitch grating of the phase mask was unusable, the main hypothesis being that its period was too close to the laser writing wavelength, increasing dramatically the phase mask zeroth order that is detrimental for FBG writing, especially for through-the-coating inscription. Thus, in practice, only 23 periods were used for the experiment.

### Spectrum measurement and refractive index determination

A basic requirement of our method was that the effective refractive index needed to be precisely determined over a broad spectral range, i.e. from the visible (550 nm) to the mid-IR (4800 nm). To that purpose, four different optical spectrum analyzers (OSA) from Yokogawa (AQ6373B, AQ6370C, AQ6376 and AQ6377) and three SC sources with different spectral coverages (400–2400 nm, 2000–3900 nm and 2800–5500 nm) were used. Measurements from the visible up to around 2 µm were taken in transmission and reflection with 50:50 fiber couplers, while spectrum beyond 2 µm were taken in transmission only due to the unavailability of such coupler. The wavelength accuracy of the four OSAs ranges from 0.02 to 0.5 nm. While the error on the determination of the effective refractive index don’t exceed 1.5 × 10^–4^ when using the visible and near-IR OSAs, the error can reach 4.5 × 10^–4^ with the mid-IR OSAs. To reach the best wavelength accuracy of our OSAs, their spectral response was calibrated with respect to the central wavelength of a He–Ne laser for the visible and near-IR OSAs (AQ6373B, AQ6370C), and according to HITRAN database at 1.3 µm (AQ6370C), 1.9 µm (AQ6376) and 2.7 µm (AQ6376, AQ6377) with absorption lines of water vapor, and at around 4.2 µm with absorption lines of CO_2_ (AQ6377). We also took advantage of the overlapping regions between the various OSAs to double check our calibration. For example, the visible OSA was calibrated with a He–Ne laser, but its precision at longer wavelength was assured by measuring a FBG near 1160 nm and comparing it with the near-IR OSA, which was calibrated with the absorption lines of water vapor near 1.3 µm. We have also checked that the uncertainty on the refractive index measurement due to a possible ambient temperature variation is less than 5 × 10^–6^, which turns out to be negligible.

It is also important to recall that two independent factors must be considered in inferring the value of ${n}_{\mathrm{eff}}$ from the measured Bragg wavelength. First, the intrinsic dc variation of the effective refractive index ${\overline{\delta n} }_{\mathrm{eff}}$ (related to the strength and visibility of the grating^27^) is resulting in a shift of the position of the nominal Bragg wavelength ${\lambda }_{0}$ according to

$${\lambda }_{0}=2\left({n}_{\mathrm{eff}}+{\overline{\delta n} }_{\mathrm{eff}}\right)\Lambda .$$

Secondly, in order to hold the fiber in a straight position in front of the phase mask, a strain $\varepsilon$ must be applied to it which is also resulting in a shift of ${\lambda }_{0}$. In fact, during inscription, the period of the written FBG always remains equal to half that of the phase mask and is independent of the strain. However, the effective refractive index decreases due to the strain-optic coefficient of the fiber, ${P}_{e}$, leading to a measured Bragg wavelength during inscription that is lower than ${\lambda }_{0}$, namely

$${\lambda }_{W}=\left(1-{P}_{e}\varepsilon \right){\lambda }_{0}.$$

When the strain is released after inscription, the effective refractive index gets back to its original value, while the period of the written FBG contracts by an amount proportional to the initial strain applied, leading to a final Bragg wavelength that is

$${\lambda }_{F}=\left(1-\varepsilon \right){\lambda }_{0}.$$

It should be noted that the photoelastic coefficient ${P}_{e}$, whose values are scarce in the literature for fluoride glasses, did not have to be known precisely to compensate for its effect since we precisely characterized the stress response of the FBG spectral shift for each tested fiber. Since measuring one of these wavelengths (writing or final) leads to an underestimation of ${n}_{\mathrm{eff}}$, this phenomenon was effectively compensated for by monitoring both wavelengths and the stress applied on each fiber to determine ${\lambda }_{0}$. In practice, a limited amount of strain was applied on each fiber, then all FBGs were written and measured one at a time, giving ${\lambda }_{W}$ for each FBG, before removing the strain on the fiber and remeasuring each individual FBGs, giving ${\lambda }_{F}$ for each of them. Regarding the shift in ${\lambda }_{0}$ due to ${\overline{\delta n} }_{\mathrm{eff}}$, it is taken into account afterward by simulating the spectrum of each individual FBG allowing for a precise determination of the parameters of each FBG according to^27^

$$r=\frac{{\mathrm{sinh}}^{2}\left(\sqrt{{\kappa }^{2}-{\widehat{\sigma }}^{2}}L\right)}{{\mathrm{cosh}}^{2}\left(\sqrt{{\kappa }^{2}-{\widehat{\sigma }}^{2}}L\right)-\frac{{\widehat{\sigma }}^{2}}{{\kappa }^{2}}},$$

where $\kappa$ and $\widehat{\sigma }$ are defined as

$${\kappa =\frac{\pi }{\lambda }\nu {\overline{\delta n} }_{\mathrm{eff}}} \mathrm{\hspace{0.5cm}and\hspace{0.5cm}} {\widehat{\sigma }=\frac{2\pi \left({n}_{\mathrm{eff}}+{\overline{\delta n} }_{\mathrm{eff}}\right)}{\lambda }-\frac{\pi }{\Lambda }.}$$

Assuming the fringe visibility $\nu$ of the 4 mm-long FBGs is close to one, then the only unknown parameters are ${n}_{\mathrm{eff}}$ and ${\overline{\delta n} }_{\mathrm{eff}}$, which can theoretically be determined with a precision of 1 × 10^–5^ when fitting the experimental spectrum. In practice, however, the fringe visibility tends to depart from unity as ${\overline{\delta n} }_{\mathrm{eff}}$ is increasing, which is resulting in a slight underestimation of the actual ${\overline{\delta n} }_{\mathrm{eff}}$, and thus a slight overestimation of ${n}_{\mathrm{eff}}$. This effect is accentuated for stronger FBGs, so that it is preferable to inscribe weak FBGs for this characterization^19,20^. When the spectra of the FBGs are only measured in transmission due to the lack of fiber couplers (i.e. beyond 2 µm), the writing parameters are chosen such that the transmission dip of the 4 mm-long FBGs never exceed − 2 dB, corresponding to a maximum ${\overline{\delta n} }_{\mathrm{eff}}$ of 2.4 × 10^–4^. This is necessary to clearly distinguish the FBG peak from the noise background. It is expected that the maximal error on the determination of ${n}_{\mathrm{eff}}$ due to the strength of these FBGs will be in the fluoride glass fibers and won’t exceed 1 × 10^–4^, which is sufficient for the present experiment considering the maximal error of 4.5 × 10^–4^ associated with the wavelength accuracy of the OSAs. The error for the FBGs whose spectra are measured in reflection (i.e. below 2 µm) are expected to be lower since they don’t need to be written as strong.

## Results and discussion

**Figure 1:**
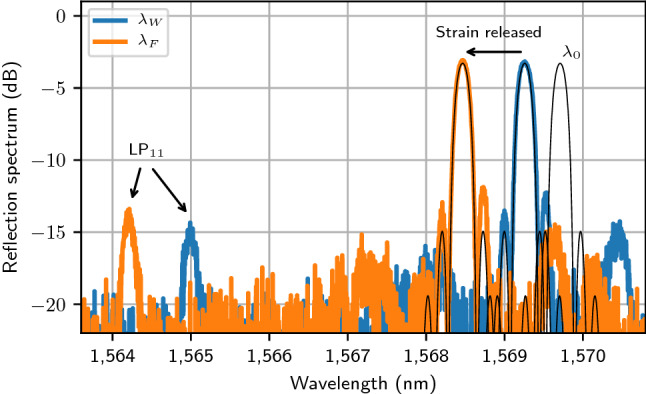
Measured FBG reflection spectrum written at the second Bragg order, with a period of 2100.0 nm, in the 7.5/125 InF_3_ fiber. The Bragg wavelength during writing, ${\lambda }_{W}$, and that obtained once the strain is released, ${\lambda }_{F}$, are both lying below ${\lambda }_{0}.$ A second peak, corresponding to the LP_11_ mode, is also present at a shorter wavelength.

**Figure 2:**
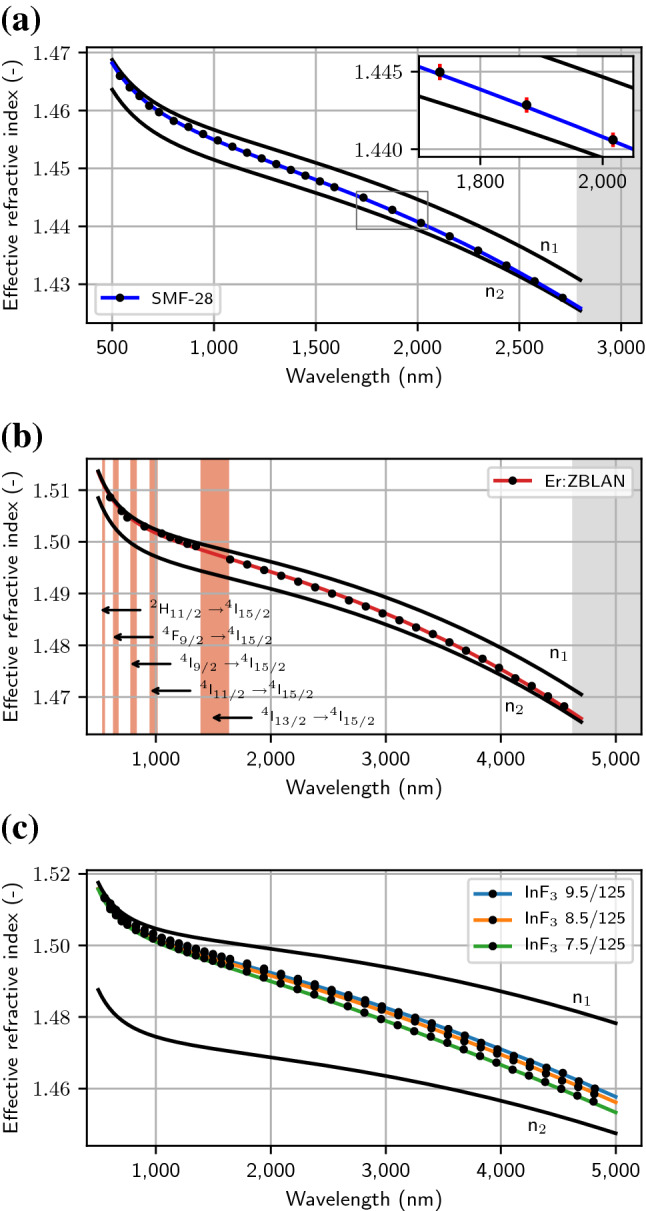
The measured effective refractive index of (**a**) the SMF-28, (**b**) the Er:ZBLAN fiber and (**c**) the three fluoroindate fibers are shown as black dots. The refractive indices of the cladding, ${n}_{2}$, are calculated from the coefficients for the two-term Sellmeier equation for each host material, listed in Table 2, while the refractive indices of the core, ${n}_{1}$, and the effective refractive indices of each fiber are calculated from their parameters listed in Table 1 by solving the characteristic equation for the weakly-guiding fiber. The limit of transparency of each fiber is shown in gray, while the absorption bands of erbium are shown in red. The inset in (**a**) shows the maximum error on the determination of the effective refractive index, since they are too small to be visible on the main figures. The error for the Er:ZBLAN fiber and the three fluoroindate fibers is the same and are not shown to not overload the figures.

The method was applied to the fibers listed in Table 1. The SMF-28, a standard silica fiber made by Corning, was chosen to validate the method, in part due to its well-known parameters, its batch-to-batch consistency and for the ease of writing FBGs in silica fibers compared to fluoride fibers. Four fluoride glass fibers, all made by *Le Verre Fluoré*, were also chosen for this demonstration: a heavily-erbium-doped ZBLAN fiber commonly used in high power 2.8 μm fiber lasers, and three undoped fluoroindate fibers used for SC generation in the mid-IR. FBGs were written through the coating of each fiber up to either the limit of their transparency (SMF-28 and Er:ZBLAN), or up to the last grating of the phase mask (InF_3_). Moreover, to extend the range of wavelength toward the shorter wavelengths for which the effective refractive index is to be determined, the second and third diffraction orders of the FBGs were also measured over the spectral ranges 800–1570 nm and 535–750 nm, respectively. For example, the reflection spectrum of the second diffraction order of a FBG written in the 7.5/125 InF_3_ fiber with a period of 2100.0 nm is shown in Fig. 1. The spectrum in blue is the spectrum after inscription, with the fiber still under tension (${\lambda }_{W}$), while the spectrum in orange is the spectrum of the same FBG once the strain is released (${\lambda }_{F}$). These peaks are both associated with the fundamental LP_01_ mode. It is interesting to note the presence of another peak in the range 1564–1565 nm which correspond to the LP_11_ mode since the fiber is multimode at this wavelength. Based on the approach detailed above, both of these wavelengths (${\lambda }_{W}$ and ${\lambda }_{F}$), in combination with the strain initially applied on the fiber, are used to determine the position of the nominal Bragg wavelength ${\lambda }_{0}$. Also, the simulation of the spectrum allows the determination of the strength of the FBG and its compensation to precisely retrieve the effective refractive index of the fiber at this wavelength. This was done for each individual FBG written in each fiber listed in Table 1, i.e. 172 times, such that the effective refractive indices of each fiber could be determined from the measured Bragg wavelengths. They are presented as black dots in Fig. 2a–c. Note that the error bars associated with them are smaller than the size of the dots for all wavelengths and are not visible on the figure. The inset in Fig. 2a was added in an attempt to show their size at the wavelengths at which they are the highest, dominated by the wavelength accuracy of the OSAs. The corresponding error bars for the Er:ZBLAN fiber and the three fluoroindate fibers are the same as for the SMF-28 and are not shown to not overload the figures.

**Table 1:** Fibers used in the experiment. The labels ZrF_4_ and InF_3_ refer to the main constituent of the corresponding multicomponent glasses.

Fiber name	Manufacturer	Host material	Size (μm)	NA (–)	${\lambda }_{C}$ (μm)
SMF-28	Corning	SiO_2_	8.2/125	0.14	1.310
ZFG DC [2.5] (ErF3 70000) 15/240*260/290	Le Verre Fluoré	ZrF_4_	15/240 × 260	0.125	2.5
IFG SM [2.95] 7.5/125	Le Verre Fluoré	InF_3_	7.5/125	0.30	2.95
IFG SM [3.3] 8.5/125	Le Verre Fluoré	InF_3_	8.5/125	0.30	3.3
IFG SM [3.7] 9.5/125	Le Verre Fluoré	InF_3_	9.5/125	0.30	3.7

For the SMF-28 fiber, the standard 3-term Sellmeier equation for germanosilicate glass was initially used to estimate the refractive index of the core (${n}_{1}$) and the cladding (${n}_{2}$), which were then used to calculate the expected effective refractive index for the LP_01_ mode by solving the characteristic equation for the weakly-guiding fiber, as described in detail in Ref.^28^, with good agreement, i.e. less than 1.5% of deviation over the full spectral range of measurement. However, for the fluoride glass fibers, it was more convenient to adjust the coefficients of a two-term Sellmeier equation of the form

$${n}^{2}-1=\sum_{i=1}^{2}\frac{{A}_{i}{\lambda }^{2}}{{\lambda }^{2}-{\lambda }_{i}^{2}}$$

since none of the available data found in the literature would correspond to our fibers^14,15^. In fact, the precise value of the refractive index of a fiber depends on several factors including its level of dopants, its geometry and its drawing conditions, the latter alone being able to account for a refractive index decrease as large as 8 × 10^–3^ in fluoride fibers, compare to the corresponding preform material^16^. For this reason, for each glass material, including the silica fiber used to validate the procedure, the refractive index of the cladding was adjusted with a two-term Sellmeier equation and the refractive index of the core was determined from the fiber’s numerical aperture, such that the calculated ${n}_{\mathrm{eff}}$ matches with the measured one. The inferred Sellmeier coefficients for each glass material are listed in Table 2.

**Table 2:** Sellmeier coefficients for the cladding glasses of the three glass materials.

Glass material	${A}_{1}$ (-)	${A}_{2}$ (-)	${\lambda }_{1}$ (μm)	${\lambda }_{2}$ (μm)
SiO_2_	1.1064	0.7250	0.091	9.11
ZrF_4_	1.2350	0.7520	0.090	14.47
InF_3_	1.1650	1.1570	0.100	20.90

Interestingly, the asymptotical behavior of the effective refractive index at short and long wavelengths for each fiber is consistent with what one would expect from mode confinement considerations. At short wavelengths, the mode is expected to be tightly confined in the core, such that ${n}_{\mathrm{eff}}\to {n}_{1}$, while at long wavelengths, the mode is expected to be loosely confined, such that ${n}_{\mathrm{eff}}\to {n}_{2}$. Further below the cutoff wavelength, which are listed in Table 1, other distinct peaks started appearing, corresponding to higher order modes. They were not considered in the present report but could definitively be useful in the future for determining and validating parameters of interest of these fibers, such as their core and cladding refractive indices, their numerical aperture, their cutoff wavelength and even their core diameter.

The dispersion of the fiber, $D$, can be numerically derived from the effective refractive index^28^:

$$D=\frac{-\lambda }{c}\frac{{\mathrm{d}}^{2}{n}_{\mathrm{eff}}}{{\mathrm{d}\lambda }^{2}}.$$

**Figure 3:**
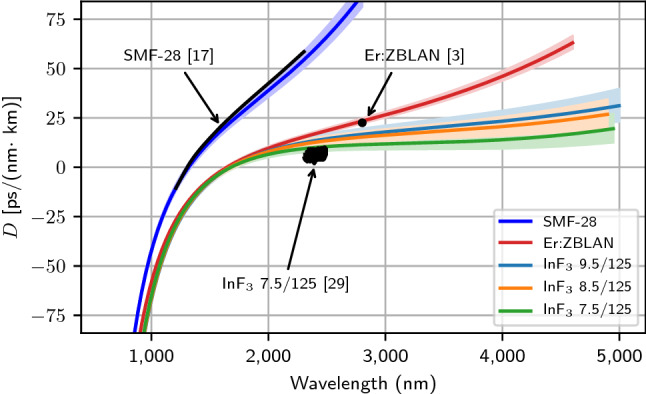
Calculated dispersion, $D$, of the fibers listed in Table 1 from the measured effective refractive indices of Fig. 2. Three measurements of the dispersion of these fibers reported in the literature are also shown for comparison. The error associated with the determination of the dispersion of each fiber is shown in a paler color around each curve.

Dispersion is thus simply obtained via a second order numerical derivation of the ${n}_{\mathrm{eff}}$ curve of Fig. 2. Although it was possible to conveniently fit the experimental data for ${n}_{\mathrm{eff}}$ with different types of functions, such as polynomials and rational functions, it appeared to us that the dispersion curves derived from them would generally tend to diverge, especially at both edges of the spectral window. In fact, we found out that a two-term Sellmeier equation was the most natural or physically sound choice for fitting the refractive index of the cladding of each glass. In fact, the dispersion curves derived from such Sellmeier equations appeared to present a more physically reliable behavior over the whole spectral range. The results are shown in Fig. 3, where the dispersion of each fiber is determined up to the last measured FBG. While the SMF-28’s manufacturer gives a zero-dispersion wavelength (ZDW) ranging between 1302 nm and 1322 nm, our measurements give a ZDW of 1318 ± 19 nm and the curve is in exceptional agreement with the manufacturer curve in the range 1200–1600 nm, while still being in good agreement up to 2300 nm, as measured by Ciąćka et al.^17^. The error on the ZDW is estimated by changing the parameters of the two-term Sellmeier equation until the calculated effective refractive index shown in Fig. 1 starts to deviate, visually, from the measured data. Regarding the fluoride glass fibers, their dispersion is not so well known, making it hard to make a valid comparison. Nonetheless, the Er:ZBLAN fiber and the InF_3_ fibers exhibit ZDWs of 1646 ± 15 nm and 1680 ± 37 nm according to our measurement, respectively, which is close to the values of 1650 nm and 1710 nm that were reported for similar fibers^1,7^. Moreover, measurements of the dispersion of the Er:ZBLAN fiber around 2.8 μm and a similar 7.5/125 InF_3_ fiber reported in the literature are close to our determined dispersion^3,29^. Finally, since there is no direct analytical formula to retrieve the dispersion curves from Fig. 3, a rational function of the form $D\left(\lambda \right)=({p}_{1}{\lambda }^{3}+{p}_{2}{\lambda }^{2}+{p}_{3}\lambda +{p}_{4})/({\lambda }^{2}+{q}_{1}\lambda +{q}_{2})$ was fitted on each one. For each tested fiber, their coefficients are listed in Table 3.

**Table 3:** Coefficient for the estimation of the fibers’ dispersion with a rational function of the form $D\left(\lambda \right)=({p}_{1}{\lambda }^{3}+{p}_{2}{\lambda }^{2}+{p}_{3}\lambda +{p}_{4})/({\lambda }^{2}+{q}_{1}\lambda +{q}_{2})$.

Fiber name	${p}_{1}$ (μm^−3^)	${p}_{2}$ (μm^−2^)	${p}_{3}$ (μm^−1^)	${p}_{4}$ (–)	${q}_{1}$ (μm^−1^)	${q}_{2}$ (–)	Standard deviation (ps/(nm km))
SMF-28	150.5	− 549.6	918.8	− 600.0	1.374	− 0.4602	0.39
Er:ZBLAN	37.82	− 172.6	375.9	− 318.6	0.5789	− 0.2506	0.67
InF_3_ 7.5/125	8.639	− 53.60	182.8	− 196.3	− 0.0418	− 0.0599	0.52
InF_3_ 8.5/125	8.338	− 36.09	130.3	− 155.4	− 0.1919	− 0.0125	0.41
InF_3_ 9.5/125	8.732	− 32.51	115.9	− 143.5	− 0.2369	0.0018	0.37

The dispersion inferred here is limited by a few factors. First, the refractive indices are approximated by a Sellmeier equation with two terms, i.e. one in the UV and one in the mid-IR. In reality, fluoride fibers generally have as much as five or six different glass constituents, leading to multiple resonances in the UV and the far mid-IR. Moreover, the core has a different composition than the cladding in order to increase its refractive index, leading to refractive indices that can have slightly different shapes, and a numerical aperture that slightly varies along the broad spectrum. The overall contribution of these multiple resonances was not accounted for here, but might have a small impact on the shape of the dispersion near the edges of the different curves, i.e. in the visible and the mid-IR.

It is also interesting to note that, even if in this experiment only the Bragg wavelengths of the LP_01_ mode in the fibers were measured, other resonances were observed whenever the fibers were multimode. In the future, the Bragg wavelengths associated with these higher order modes could be used to increase the accuracy of the fitted refractive index for the core and the cladding by forcing the calculated effective refractive index for each mode to pass through these values too. In fact, with these values and a better post-processing of the data, we believe that all of the fiber parameters, such as its core and cladding refractive indices, its numerical aperture, its core diameter, its cutoff wavelength, etc., could be inferred with high precision.

Finally, it is worth mentioning that the proposed method is destructive, in the sense that FBGs need to be written over 12 cm of the fiber under test. Since another 10–20 cm is currently needed on both sides of the FBG to couple the probing source and for guiding the probing signal to the characterization setup, we estimate that a length of about 30–50 cm is needed to characterize the dispersive properties of a fiber sample.

## Conclusion

In summary, this paper has reported a method to accurately determine the effective refractive index and the dispersion of various optical fibers over a large bandwidth by writing arrays of FBGs using femtosecond pulses. This method was applied to silica- and fluoride-based optical fibers over almost their entire transmission range and has the potential to be extended to characterize the dispersion of other exotic fibers such as those based on tellurite and chalcogenide glass, and to characterize other optical and physical parameters of such fibers, such as their cutoff wavelength and their numerical aperture by measuring the Bragg wavelength associated with higher order modes propagating in the fiber.

## Data Availability

The datasets generated during and/or analyzed during the current study are available from the corresponding author on reasonable request.
